# Resveratrol Treatment after Status Epilepticus Restrains Neurodegeneration and Abnormal Neurogenesis with Suppression of Oxidative Stress and Inflammation

**DOI:** 10.1038/srep17807

**Published:** 2015-12-07

**Authors:** Vikas Mishra, Bing Shuai, Maheedhar Kodali, Geetha A. Shetty, Bharathi Hattiangady, Xiaolan Rao, Ashok K. Shetty

**Affiliations:** 1 Institute for Regenerative Medicine, Texas A & M Health Science Center College of Medicine at Scott & White, Temple, Texas, USA; 2 Research Service, Olin E. Teague Veterans’ Affairs Medical Center, Central Texas Veterans Health Care System, Temple, Texas, USA; 3 Department of Molecular and Cellular Medicine, Texas A&M Health Science Center College of Medicine, College Station, Texas, USA

## Abstract

Antiepileptic drug therapy, though beneficial for restraining seizures, cannot thwart status epilepticus (SE) induced neurodegeneration or down-stream detrimental changes. We investigated the efficacy of resveratrol (RESV) for preventing SE-induced neurodegeneration, abnormal neurogenesis, oxidative stress and inflammation in the hippocampus. We induced SE in young rats and treated with either vehicle or RESV, commencing an hour after SE induction and continuing every hour for three-hours on SE day and twice daily thereafter for 3 days. Seizures were terminated in both groups two-hours after SE with a diazepam injection. In contrast to the vehicle-treated group, the hippocampus of animals receiving RESV during and after SE presented no loss of glutamatergic neurons in hippocampal cell layers, diminished loss of inhibitory interneurons expressing parvalbumin, somatostatin and neuropeptide Y in the dentate gyrus, reduced aberrant neurogenesis with preservation of reelin + interneurons, lowered concentration of oxidative stress byproduct malondialdehyde and pro-inflammatory cytokine tumor necrosis factor-alpha, normalized expression of oxidative stress responsive genes and diminished numbers of activated microglia. Thus, 4 days of RESV treatment after SE is efficacious for thwarting glutamatergic neuron degeneration, alleviating interneuron loss and abnormal neurogenesis, and suppressing oxidative stress and inflammation. These results have implications for restraining SE-induced chronic temporal lobe epilepsy.

Multiple conditions including head trauma, stroke and Alzheimer’s disease can trigger status epilepticus (SE). Hippocampus is highly susceptible to SE where a cascade of morphological and functional changes collectively referred to as epileptogenesis occur over weeks and months after SE and cause temporal lobe epilepsy (TLE), typified by spontaneous recurrent seizures (SRS), and cognitive and mood dysfunction associated with declined neurogenesis^1^,^2^,^3^,^4^,^5^,^6^. In the realm of SRS occurring in the chronic phase after SE, early changes such as loss of subclasses of gamma-amino butyric acid (GABA)-ergic interneurons^3^, increased oxidative stress, inflammation characterized by reactive astrocytes and activated microglia^7^,^8^ and abnormal neurogenesis exemplified by anomalous migration of newly born neurons into the dentate hilus and the molecular layer have received great interest^9^,^10^,^11^. On the other hand, memory and mood impairments in the chronic phase after SE have been attributed to declined neurogenesis and loss of glutamatergic neurons in the hippocampus^2^,^12^,^13^,^14^. Antiepileptic drug (AED) therapy can stop SE in most instances but cannot adequately suppress SE-induced early detrimental changes described above^5^,^15^,^16^,^17^. Because these changes contribute to epileptogenesis, AED therapy has mostly failed to prevent the evolution of SE into chronic TLE. Hence, an ideal neuroprotective strategy for SE should be capable of restraining glutamatergic and GABA-ergic neuron loss, oxidative stress, inflammation and aberrant neurogenesis. In this context, compounds and drugs having neuroprotective and/or antiepileptogenic properties are ideal for preventing SE-induced chronic hippocampal dysfunction typified by SRS, and cognitive and mood impairments.

Resveratrol (RESV), a polyphenol found abundantly in the skin of red grapes, appears to meet the above criteria as it can mediate a wide range of biological activities with no side effects^18^,^19^,^20^,^21^. The properties of RESV particularly relevant for neuroprotection and anti-epileptogenesis after SE include its ability for crossing the blood-brain barrier after systemic administration^22^, and diminishing oxidative stress^23^, apoptotic and necrotic cell death^24^ and neuroinflammation^20^,^25^,^26^. Studies in neurological disease models have also suggested that RESV is a potent neuroprotective compound^27^,^28^,^29^,^30^. Moreover, RESV administration prior to SE induction or after focal injury can restrain neuron loss and oxidative stress^22^,^26^,^31^,^32^,^33^. However, the efficacy of RESV administration commencing after the onset of full-blown SE is unknown. Therefore, using a well-established kainate model of SE, we examined the effects of RESV treatment commencing an hour after SE for easing glutamatergic and GABA-ergic neuron loss, oxidative stress, inflammation and abnormal neurogenesis in the hippocampus, using immunohistochemical, biochemical and molecular biological methods and stereological cell counts.

## Results

**Figure 1:**
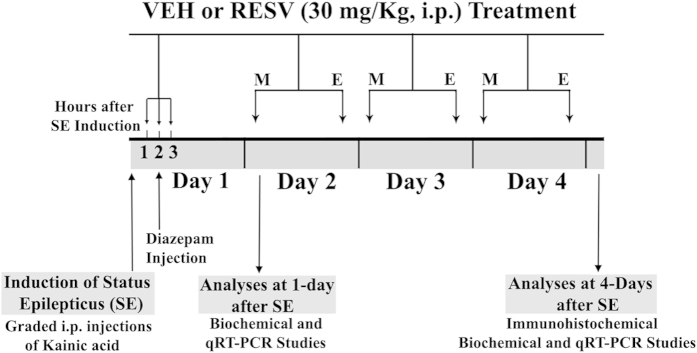
A schematic showing the experimental design and the time-line of vehicle (VEH) or resveratrol (RESV) treatment. Status epilepticus (SE) was first induced in Fischer 344 rats through graded intraperitoneal injections of Kainic acid (KA). Animals received hourly injections of VEH or RESV for three hours on the day of SE (Day 1), which commenced an hour after the onset of SE. In the following 3 days (days 2–4), animals received twice daily (morning and evening) injections of VEH or RESV. The behavioral seizures in both VEH and RESV treated groups were terminated 2 hours after the induction of SE through an intraperitoneal injection of diazepam (5 mg/Kg). Subgroups of animals were euthanized 1 and 4 days after SE along with age-matched naïve control animals and tissues harvested for biochemical and molecular biological studies. Additional subgroups of animals were euthanized 4 days after SE via intracardiac perfusions for various immunohistochemical studies. M, morning; E, evening.

The time-line of experiments, and the vehicle (VEH), RESV and diazepam treatment regimen employed after SE onset, are illustrated in Fig. 1. Status epilepticus was induced in young adult rats through graded intraperitoneal injections of kainic acid (KA), as detailed in our previous studies^12^,^34^,^35^,^36^. Additional details on procedures and animal numbers utilized for various analyses are available in “Methods” section. From here onwards, animals receiving VEH during and after SE, and animals receiving RESV during and after SE, will be referred to as “SE-VEH animals” and “SE-RESV animals” respectively.

### RESV treatment after the induction of SE did not affect behavioral seizures

The behavioral activity of animals that received graded intraperitoneal KA injections was carefully observed. In the first hour after SE onset, rats assigned to VEH and RESV groups displayed comparable episodes of stages IV and V seizures, typified by bilateral forelimb clonus or bilateral forelimb clonus with rearing and falling (SE-VEH animals, Mean ± SEM: 5.9 ± 1.4; SE-RESV animals, 6.4 ± 1.2, p > 0.05, n = 8–9). Treatment with VEH or RESV did not affect seizures in the second hour after SE, as animals in both groups exhibited similar numbers of stages IV–V seizures following the treatment (SE-VEH animals, 2.2 ± 0.9; SE-RESV animals, 2.5 ± 0.8, p > 0.05, n = 8–9). Thus, RESV treatment did not seem to affect the ongoing seizures in the second hour after SE onset. Furthermore, a subcutaneous injection of diazepam given two hours after SE onset terminated stages III-V behavioral seizures within 10–15 minutes of injection in both VEH and RESV treated groups.

### RESV administration protected hippocampal glutamatergic neurons from SE-induced death

**Figure 2:**
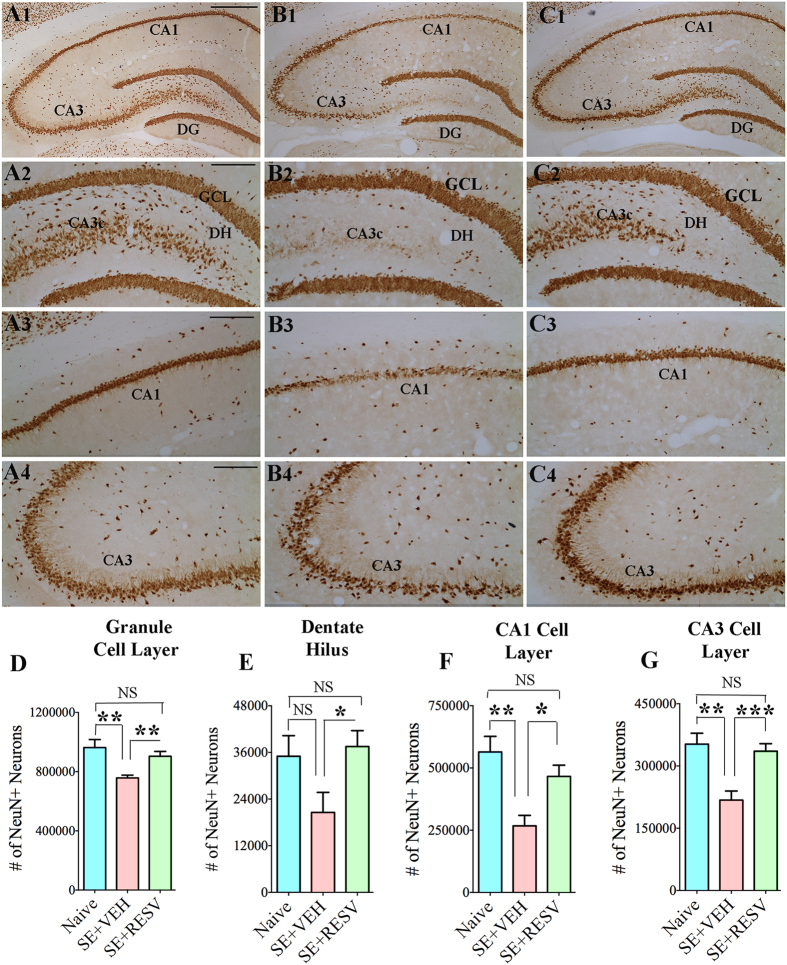
Resveratrol (RESV) treatment after status epilepticus (SE) greatly restrained neurodegeneration in the dentate hilus (DH), the granule cell layer (GCL) and the hippocampal CA1 and CA3 pyramidal cell layers. A1, B1 and C1 show neuron-specific nuclear antigen (NeuN) immunostaining of the hippocampus in a naïve control rat (**A1**) and rats that received vehicle (VEH; B1) or RESV (**C1**) after SE. Figures A2-A4, B2-B4 and C2-C4 illustrate enlarged view of the dentate gyrus (**A2,B2C2**), CA1 subfield (**A3,B3,C3**) and CA3 subfield (**A4,B4,C4**) from A1, B1 and C1. Scale bar, A1, B1 and C1, 500 μm; A2-A4, B2-B4 and C2-C4, 200 μm. Bar charts compare numbers of NeuN + neurons in different cells layers of the hippocampus between naïve control rats and rats that received VEH or RESV after SE. Note that, in comparison to naïve control rats, rats receiving VEH after SE display clearly reduced numbers of neurons in the DH (**D**), the granule cell layer (GCL; **E**) and the CA1 and CA3 pyramidal cell layers (**F,G**). Contrastingly, in rats receiving RESV during and after SE, numbers of neurons in these cell layers remain comparable to those in naïve control rats (**D–G**). *p < 0.05; **p < 0.01; ***p < 0.001; NS, not significant.

Immunostaining of brain sections through the hippocampus for NeuN visualized neurons in different cell layers of the hippocampus (Fig. 2). The overall density of neurons in the DH, GCL, and CA1 and CA3 cell layers of the hippocampus appeared clearly reduced in SE-VEH animals in comparison to age-matched naive control rats (Fig. 2(A1–B4)). In contrast, SE-RESV animals appeared to have similar density of neurons as naive control rats and greater density of neurons than SE-VEH animals (Fig. 2(C1–C4)). Stereological quantification of NeuN + neurons in different cell layers of the hippocampus confirmed considerable loss of neurons in SE-VEH animals and no significant loss of neurons in rats receiving RESV (Fig. 2(D–G)). The overall loss in SE-VEH animals was 21% in the GCL (p < 0.01), 41% in the DH (p > 0.05), 53% in the CA1 cell layer (p < 0.01), and 38% in the CA3 cell layer (p < 0.01). In contrast, SE-RESV animals displayed similar numbers of neurons as naive control rats in the GCL, DH, CA1 and CA3 pyramidal cell layers (p > 0.05, Fig. 2(D–G)). This quantification also revealed that SE-RESV animals displayed greater numbers of surviving neurons in GCL, DH, CA1 and CA3 cell layers than SE-VEH animals (p < 0.05-0.001, Fig. 2(D–G)). Thus, RESV treatment notably protected glutamatergic neurons in hippocampal cell layers from undergoing SE-induced death.

### RESV treatment diminished SE-induced loss of subclasses of GABA-ergic interneurons expressing PV, SS and NPY

**Figure 3:**
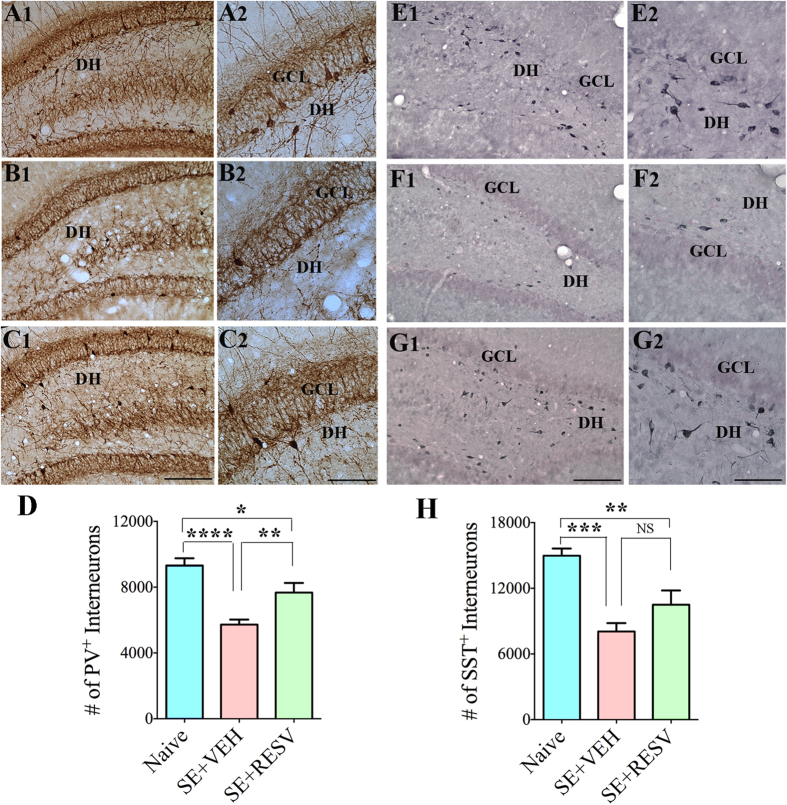
Resveratrol (RESV) treatment after status epilepticus (SE) lowered the loss of interneurons expressing parvalbumin (PV) and somatostatin (SST) in the dentate gyrus (DG). Figures A1, B1 and C1 show the distribution of PV + interneurons in the DG from a naïve control rat (**A1**) and rats that received vehicle (VEH; **B1**) or RESV (**C1**) after SE. Figures A2, B2 and C2 are magnified views of regions from A1, B1 and C1. Figures E1, F1 and G1 show the distribution of SST + interneurons in the DG from a naïve control rat (**E1**) and rats that received VEH (**F1**) or RESV (**C1**) after SE. Figures E2, F2 and G2 are magnified views of regions from E1, F1 and G1. DH, Dentate hilus; GCL, granule cell layer. Scale bar: A1, B1, C1, E1, F1 and G1, 200 μm; A2, B2, C2, E2, F2 and G2, 100 μm. Bar charts compare numbers of interneurons positive for PV (**D**) and SST (**H**) in the DG between naïve control rats and rats that received VEH or RESV after SE. Note that, in comparison to naïve control rats, rats receiving VEH after SE display considerable reductions in numbers of PV + and SST + interneurons. In contrast, rats receiving RESV during and after SE display moderate loss of PV + and SST + interneuron numbers and hence exhibit greater numbers surviving PV + and SST + interneurons than rats receiving VEH after SE. *p < 0.05; **p < 0.01; ***p < 0.001; ****p < 0.0001.

**Figure 4:**
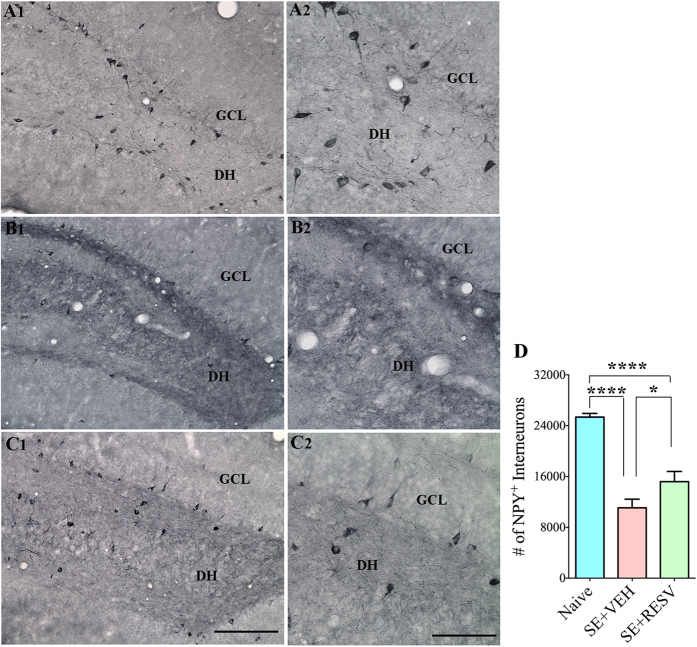
Resveratrol (RESV) treatment after status epilepticus (SE) moderated the loss of interneurons expressing neuropeptide Y (NPY) in the dentate gyrus (DG). Figures A1, B1 and C1 show the distribution of NPY + interneurons in the DG from a naïve control rat (**A1**) and rats that received vehicle (VEH; **B1**) or RESV (**C1**) after SE. Figures A2, B2 and C2 are magnified views of regions from A1, B1 and C1. DH, Dentate hilus; GCL, granule cell layer. Scale bar: (**A1,B1,C1**) 200 μm; (**A2,B2,C2**) 100 μm. Bar chart compares the numbers of interneurons positive for NPY in the DG between naïve control rats and rats that received VEH or RESV after SE. Note that, in comparison to naïve control rats, rats receiving VEH after SE display a major reduction in numbers of NPY + interneurons. In contrast, rats receiving RESV during and after SE display reduced loss of NPY + interneurons and hence show greater numbers surviving NPY + interneurons than rats receiving VEH after SE. DH, Dentate hilus; GCL, granule cell layer. *p < 0.05; ****p < 0.0001.

We examined the efficacy of RESV treatment to protect different subclasses of hippocampal GABA-ergic interneurons in the dentate gyrus (DG) against SE-induced loss. In comparison to naive control animals, SE-VEH animals displayed an apparently reduced density of interneurons expressing the calcium binding protein PV (Fig. 3(A1–C2)), and neuropeptides SST (Fig. 3(E1–G2)) and NPY (Fig. 4(A1–C2)) in the DG. Stereological quantification confirmed considerable loss of these interneurons. The reductions were 39% for PV + interneurons (p < 0.0001, Fig. 3(D)), 46% for SST + interneurons (p < 0.001, Fig. 3(H)), and 56% for NPY + interneurons (p < 0.0001, Fig. 4(D)). In contrast, SE-RESV animals showed reduced loss of interneurons in the DG (Fig. 3(D,H) and 4[D]). In comparison to naive control animals, the reductions were 18% for PV + interneurons (p < 0.05), 30% for SST + interneurons (p < 0.01) and 40% for NPY + interneurons (p < 0.0001). Additional analyses revealed that, in comparison to SE-VEH animals, the DG of SE-RESV animals contained greater numbers of interneurons expressing PV (34% more, p < 0.01), SST (30% more, p > 0.05), and NPY (37% more, p < 0.05, Figs 3(D,H) and 4(D)). Thus, RESV administration during and after SE considerably restrained the loss of various subclasses of GABA-ergic interneurons in the DG of the hippocampus.

### RESV administration during and after SE restrained abnormal hippocampal neurogenesis with preservation of interneurons expressing reelin

**Figure 5:**
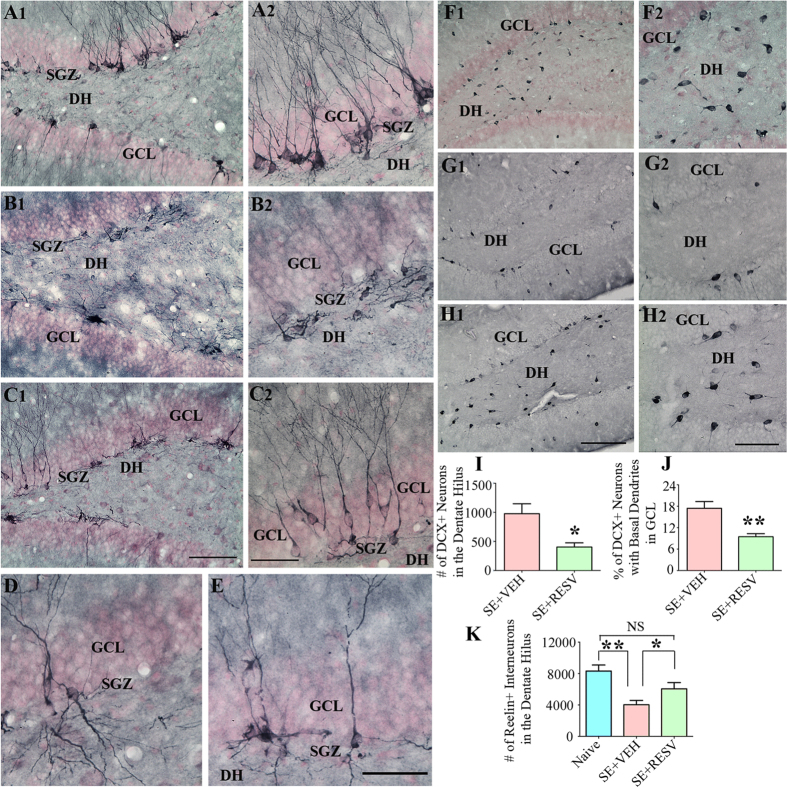
Resveratrol (RESV) treatment after status epilepticus (SE) curbed abnormal hippocampal neurogenesis with preservation of interneurons expressing reelin. Figures A1, B1 and C1 show the distribution of doublecortin (DCX) positive newly born neurons in the dentate gyrus (DG) subgranular zone (SGZ), granule cell layer (GCL) and hilus (DH) from a naïve control rat (**A1**) and rats that received vehicle (VEH; **B1**) or RESV (**C1**) after SE. A2, B2 and C2 are magnified views of regions from A1, B1 and C1 showing the morphology of DCX + neurons. Note that, DCX + neurons are restricted to the SGZ-GCL with dendrites projecting into the molecular layer in the DG of naive control rat (**A1, A2**) whereas, in the DG of rat receiving VEH after SE, DCX + neurons have moved mostly into the DH (**B1, B2**), implying abnormal migration. In contrast, in the DG of rat receiving RESV during and after SE, majority of DCX + neurons remained in the SGZ-GCL, depicting minimal abnormal migration. Figures D and E show DCX + newly born granule cells exhibiting basal dendrites projecting into the DH in rats that received VEH (**D**) or RESV (**E**) after SE. Figures F1, G1 and H1 show the distribution of reelin + interneurons in the DH from a naïve control rat (**F1**) and rats that received VEH (**G1**) or RESV (**H1**) after SE. F2, G2 and H2 are magnified views of regions from F1, G1 and H1. Scale bar: A, B1, C1, F1, G1 and H1, 200 μm; A2, B2, C2, F2, G2 and H2, 100 μm; D and E, 50 μm. Bar charts in I and J compare the numbers of DCX + neurons in the DH (**I**) and percentages of DCX + neurons in the GCL exhibiting basal dendrites (**J**). Rats receiving RESV during and after SE display reduced numbers of DCX + neurons into the DH and reduced percentages of DCX + neurons exhibiting basal dendrites. Bar chart in K compares the numbers of reelin + interneurons between groups. Rats receiving VEH after SE show considerable loss of reelin + interneurons in comparison to the other two groups, whereas rats receiving RESV during and after SE demonstrate no loss of reelin + interneurons. *p < 0.05; **p < 0.01; NS, not significant.

Newly born DCX + neurons incorporate solely into the SGZ-GCL in the DG of naive control animals (Fig. 5(A1). Hence, newly born DCX + neurons were either absent or rarely seen in the DH of these animals. In contrast, in SE-VEH animals, considerable numbers of newly born DCX + neurons migrated abnormally into the DH (Fig. 5(B1), which is a consistent effect of SE found in virtually all animal models of SE examined so far^1^,^2^,^9^,^37^. However, animals receiving RESV treatment after SE displayed considerably reduced numbers of newly born DCX + neurons in the DH (Fig. 5(C1, 59% reduction, p < 0.05), suggesting that RESV administration during and after SE onset can restrain the abnormal migration of newly born neurons. Examination of the extent of normal neurogenesis in the SGZ-GCL region did not show any apparent differences between SE-VEH and SE-RESV animals however.

We also examined and quantified the occurrence of basal dendrites projecting into the dentate hilus from DCX + newly born neurons that incorporated into the SGZ and GCL area with one or more major dendrites projecting into the dentate molecular layer (i.e. from relatively mature DCX + newly born neurons, Fig. 5(D,E)). SE-RESV animals displayed reduced percentages of DCX + neurons with basal dendrites than SE-VEH animals (Fig. 5(J), 46% reduction, p < 0.01). Thus, in comparison to SE-VEH animals, SE-RESV animals exhibited considerably reduced abnormal neurogenesis. Because reelin is involved in the appropriate migration of DCX + newly born neurons into the SGZ-GCL area and reelin is derived from subclasses of interneurons, we evaluated the number of interneurons expressing reelin in DH (Fig. 5(F1–H2)). In comparison to naive control animals, SE-VEH animals showed greatly reduced numbers of interneurons expressing reelin in the DH (52% reduction, p < 0.01, Fig. 5(K)). However, the loss of reelin + interneurons in the DH was not significant in SE-RESV animals (p > 0.05, Fig. 5(K)). Thus, RESV administration during and after SE restrained the extent of abnormal hippocampal neurogenesis through considerable protection of reelin + neurons in the DH.

### RESV treatment eased SE-induced oxidative stress

**Figure 6:**
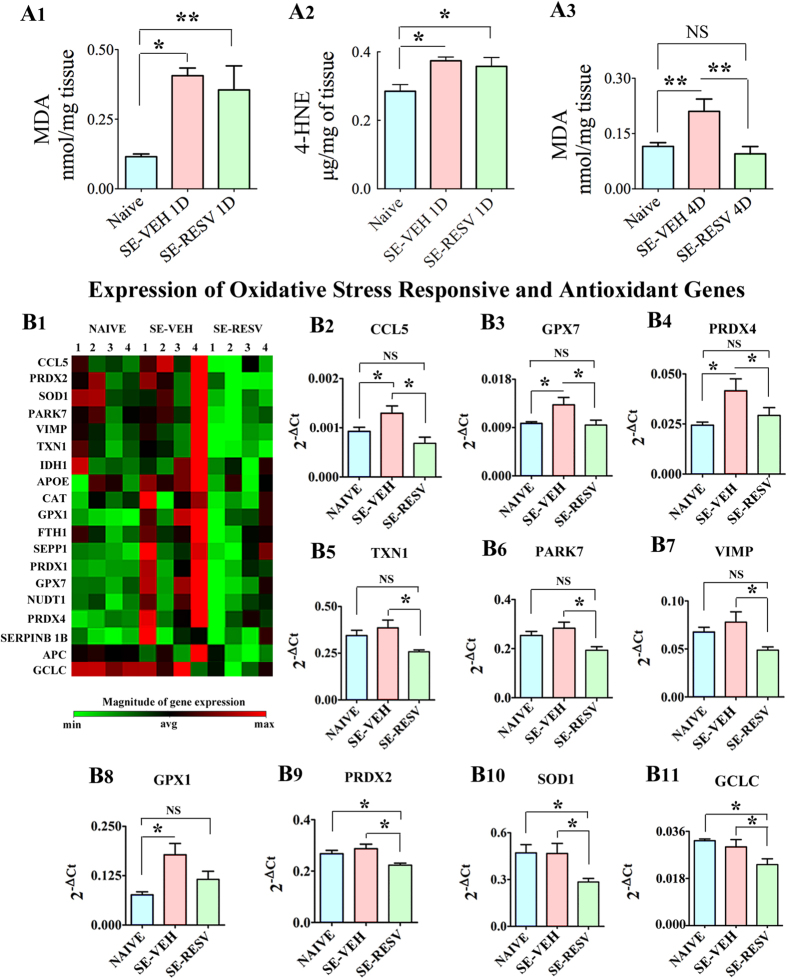
Resveratrol (RESV) treatment for 4 days normalized status epilepticus (SE) induced increased oxidative stress in the hippocampus. Bar charts in A1, A2 and A3 compare concentrations of malondialdehyde (MDA; **A1,A3**) and 4-hydroxynoneal (4-HNE; **A2**) between different groups. When measured a day after SE, rats receiving vehicle (VEH) or RESV showed increased concentration of MDA (**A1**) and 4-HNE (**A2**) in comparison to naive control rats. However, when measured 4 days after SE, MDA concentration was normalized to control levels in rats receiving RESV but remained upregulated in rats receiving VEH after SE (**A3**), implying the beneficial effects of RESV treatment for 4 days after SE. Figure B1 illustrates the expression (heat map) of select oxidative stress responsive and antioxidant genes measured through quantitative real time PCR array in the hippocampus. Note that, in comparison to their expression in naive control rats, many genes show a trend towards increased expression in rats receiving VEH after SE but normalized expression in rats receiving RESV during and after SE (**B1**), suggesting extinction of increased oxidative stress with RESV treatment. Bar charts in B2-B11 compare the expression of specific genes between the three groups. Note that, RESV treatment after SE normalized the expression of genes CCL5, GPX7, PRDX4, TXN1, PARK7, VIMP and GPX1 to control levels (**B2–B8**) and lowered the expression of genes PRDX2, SOD1 and GCLC to below control levels (**B9–B11**), implying considerable moderation of oxidative stress in the hippocampus by RESV treatment.

Both MDA (a byproduct of lipid peroxidation and an important biomarker of membrane damage) and 4-HNE (α, β-unsaturated hydroxyalkenal produced by lipid peroxidation in cells) levels were increased at 1-day after SE in VEH-treated rats, implying significantly enhanced oxidative stress after SE in the hippocampus (Fig. 6(A1). In comparison to naive control animals, the increases were 253% for MDA (p < 0.05) and 31% for 4-HNE (p < 0.05). Interestingly, RESV treatment did not significantly alter MDA and 4-HNE levels at 1-day after SE, as SE-RESV animals displayed 208% greater MDA and 25% greater 4-HNE levels than naive control animals (p < 0.05-0.01, Fig. 6(A1). However, major difference in MDA concentration emerged between SE-VEH and SE-RESV groups at 4 days after SE. While SE-VEH animals exhibited 83% greater MDA levels than naive control rats (p < 0.01), SE-RESV animals displayed levels of MDA that are highly comparable to naive control animals (p > 0.05, Fig. 6(A3)). Consequently, SE-VEH animals had 120% greater MDA concentration than SE-RESV animals at 4-days after SE (p < 0.01, Fig. 6(A3)). Thus, 4-days of RESV treatment eliminated SE-induced increased oxidative stress in the hippocampus.

### RESV treatment normalized the expression of many oxidative stress response genes

Because MDA concentration was stabilized to baseline levels with 4 days of RESV treatment after SE, we examined whether this regulation is also evident through the expression pattern of genes that respond to oxidative stress. Among 84 genes examined through qRT-PCR array, a fraction of genes showed significant differences in their expression between naive control, SE-VEH and SE-RESV animals (p < 0.05, one-way ANOVA, Fig. 6(B1–B11)): (1) CCL5—a gene encoding T-cell specific RANTES protein (C-C Motif chemokine 5) having a role in recruiting leukocytes into inflammatory sites (Fig. 6(B2)). (2) GPX7 and GPX1—genes encoding glutathione peroxidases 1 and 7, which are enzymes that couple the oxidation of reduced glutathione to the detoxification of peroxides (Fig. 6(B3). (3) PRDX4 and PRDX2—genes encoding peroxiredoxins 4 and 2, which are antioxidant enzymes involved in reducing hydrogen peroxide and alkyl hydroperoxides to water and alcohol with the use of reducing equivalents derived from thiol-containing donor molecules (Fig. 6(B4). (4) TXN1—a gene encoding thioredoxin 1, which participates in various redox reactions through the reversible oxidation of its active center dithiol to a disulfide and catalyzes dithiol-disulfide exchange reactions (Fig. 6(B5)). In addition, it plays a role in the reversible S-nitrosylation of cysteine residues in target proteins, and thereby takes part in the response to intracellular nitric oxide. (5) PARK7—a gene encoding Parkinson protein 7, which functions as a redox-sensitive chaperone (a sensor for oxidative stress) and protects neurons against oxidative stress and cell death (Fig. 6(B6)). (6) VIMP—a gene encoding VCP interacting membrane protein involved in the degradation process of misfolded endoplasmic reticulum luminal proteins (Fig. 6(B7)). (7) SOD1—a gene encoding the most abundant cytosolic superoxide dysmutase 1, which binds to molecules of copper (Cu) and zinc (Zn) to break down toxic, charged oxygen molecules called superoxide radicals (Fig. 6(B10)). (8) GCLC—a gene encoding glutamate-cysteine ligase (gamma-glutamylcysteine synthetase), which is the first rate-limiting enzyme of glutathione synthesis. Of the above genes, the expression of CCL5, GPX7, PRDX4 and GPX1 was significantly upregulated in SE-VEH animals (p < 0.05 in comparison naive control rats) but normalized to control levels in SE-RESV animals (p > 0.05) (Fig. 6(B2–B4). On the other hand, the expression of genes TXN1, PARK7 and VIMP was modestly upregulated in SE-VEH animals (p > 0.05) but RESV treatment for 4 days significantly reduced their expression (p < 0.05 in comparison to SE-VEH animals and p > 0.05 in comparison to naive controls, Fig. 6(B5). The expression of genes PRDX2, SOD1 and GCLC in SE-VEH animals remained similar to naive control rats but RESV treatment significantly reduced their expression (p < 0.05, in comparison to both SE-VEH animals and naive control rats, Fig. 6(B9–B11)).

Furthermore, though not significant statistically, several other genes responsive to oxidative stress displayed a trend towards reduced expression in SE-RESV animals in comparison to SE-VEH animals (Fig. 6(B1)). These include: (1) CAT—a gene encoding catalase, which breaks down hydrogen peroxide (H_2_O_2_) molecules into oxygen (O_2_) and water (H_2_O). (2) NUDT1—a gene encoding Nudix (Nucleoside Diphosphate Linked Moiety X) -Type Motif 1, which acts as a sanitizing enzyme for oxidized nucleotide pools to prevent cell dysfunction and death induced by oxidative stress. (3) IDH1—a gene encoding the production of isocitrate dehydrogenase 1, which converts isocitrate to 2-ketoglutarate to produce NADPH necessary for many cellular processes and protection against ROS. (4) PRDX1—a gene encoding peroxiredoxin 1 involved in reducing hydrogen peroxide. (5) SERPINB 1B—a gene encoding Ovalbumin (Serpin Peptidase Inhibitor Clade B), which inhibits the neutrophil-derived proteinases neutrophil elastase, cathepsin G, and proteinase-3 to protect tissues from damage at inflammatory sites. (6) SEPP1—a gene encoding Selenoprotein P, Plasma 1, which is believed to be responsible for some of the extracellular antioxidant defense properties of selenium. (7) FTH1—a gene encoding heavy subunit of ferritin, which stores iron in a soluble and nontoxic state. (8) APC—a gene encoding tumor suppressor adenomatous polyposis coli (APC) protein having a role in several cellular processes. (9) APOE—a gene encoding apolipoprotein E, which is a very low-density lipoprotein involved in removing cholesterol. Thus, in comparison to SE-VEH animals, SE-RESV animals displayed diminished expression of multiple genes related to oxidative stress. Because these genes typically exhibit increased expression in response to oxidative stress, the results imply normalization of oxidative stress with RESV treatment, consistent with MDA results described above.

Additionally, five genes relevant to oxidative stress showed reduced expression in SE-RESV animals in comparison to control animals (p < 0.05, data not illustrated). These include: GSTP1, which encodes glutathione s-transferase Pi 1 involved in detoxification by catalyzing the conjugation of many hydrophobic and electrophilic compounds with reduced glutathione; SOD2, which encodes mitochondrial protein superoxide dysmutase 2 that binds to the superoxide byproducts of oxidative phosphorylation and converts them to hydrogen peroxide and diatomic oxygen; GSR, which encodes glutathione reductase, a central enzyme of cellular antioxidant defense that reduces oxidized glutathione disulfide (GSSG) to the sulfhydryl form of GSH, which is an important cellular antioxidant; IFT172, which encodes an oxygen transporter, intraflagellar transport 172 Homolog; and FANCC, which encodes Fanconi anemia, complementation group C involved in DNA repair. However, the expression of these genes did not differ between SE-RESV and SE-VEH animals as well as between naive and SE-VEH animals. Thus, the expression of multiple genes that typically display upregulated expression to increased oxidative stress was either normalized to control levels or pushed to levels lower than that of controls by RESV treatment, which is suggestive of greatly diminished oxidative stress in the hippocampus of rats that received RESV after SE.

### RESV treatment after SE decreased TNF-α concentration and the numbers of activated microglia

**Figure 7:**
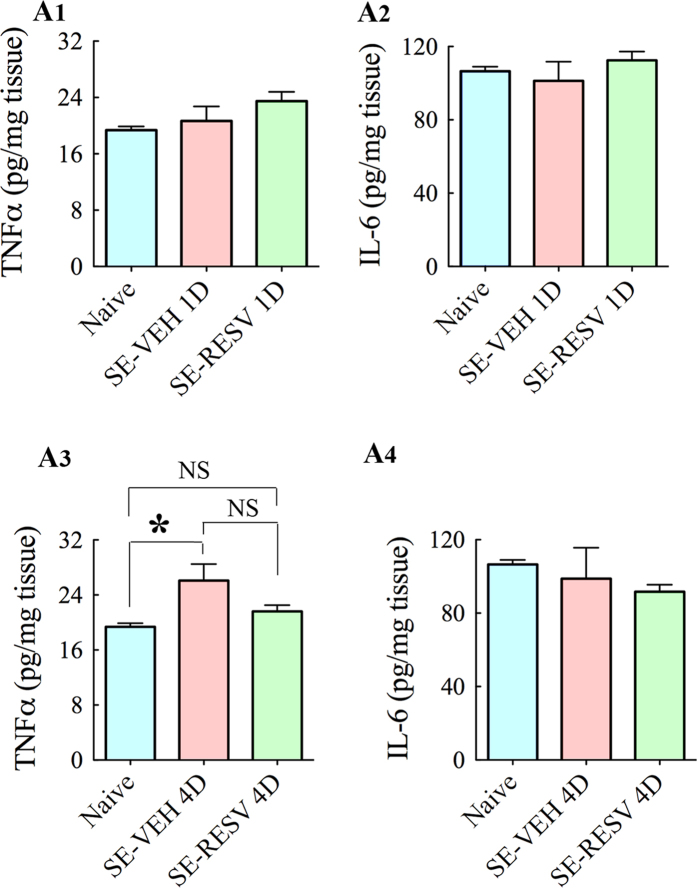
Resveratrol (RESV) treatment for 4 days normalized status epilepticus (SE) induced increased concentration of tumor necrosis factor-alpha (TNF-α) in the hippocampus. Bar charts in A1-A4 compare concentrations of pro-inflammatory cytokines TNF-α (**A1,A3**) and interleukin-6 (IL-6; **A2,A4**) between different groups. When measured a day after SE, rats receiving vehicle (VEH) or RESV showed no increase in concentrations of TNF-α (**A1**) or IL-6 (**A2**) in comparison to naive control rats. However, when measured 4 days after SE, TNF-α concentration was normalized to control levels in rats receiving RESV but was upregulated in rats receiving VEH after SE (**A3**). On the other hand, IL-6 concentration did not vary between groups even at 4 days post-SE.

**Figure 8:**
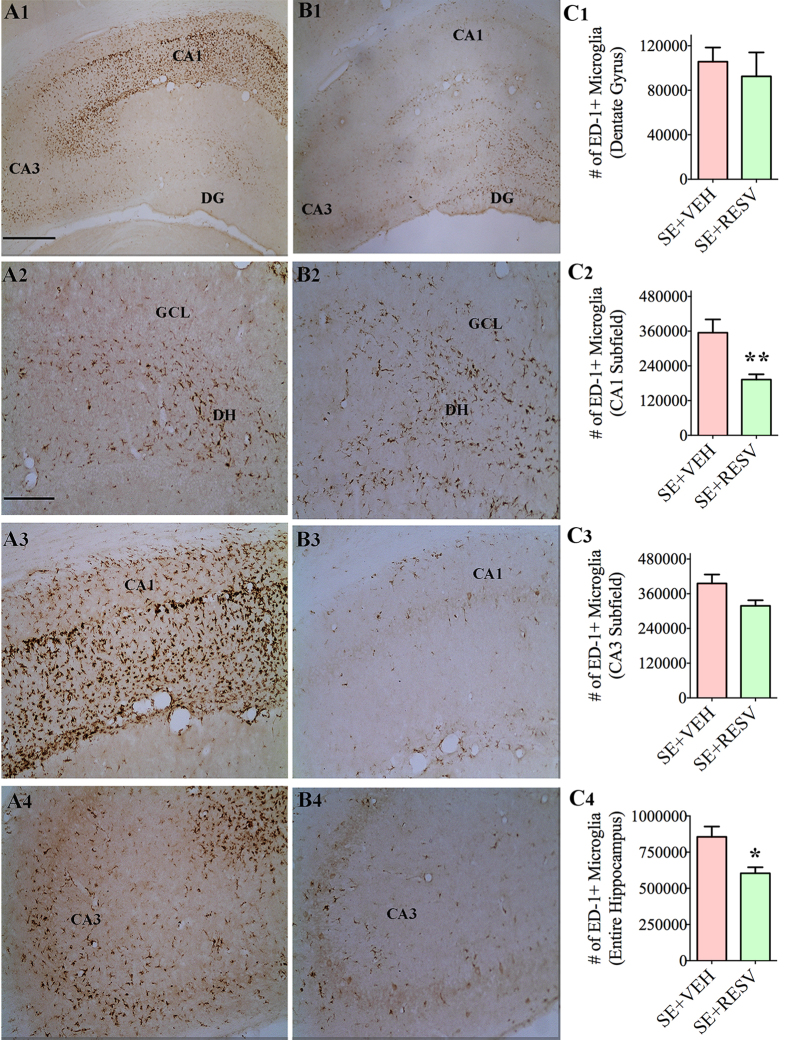
Resveratrol (RESV) treatment for 4 days reduced status epilepticus (SE) induced ED1 + activated microglia in the hippocampus. Figures A1 and B1 show the distribution of ED-1 + activated microglia in the hippocampus of rats receiving vehicle (VEH; **A1**) or RESV (**B1**) after SE. A2, A3 and A4 are magnified views of regions from A1 whereas B2, B3 and B4 are magnified views of regions from B1, showing the distribution of ED-1 + microglia in the dentate gyrus (DG; **A2,B2**), CA1 subfield (**A3,B3**) and the CA3 subfield (**A4,B4**). DH, Dentate hilus; GCL, granule cell layer. Scale bar, A1 and B1, 500 μm; A2–A4 and B2-B4, 200 μm. Bar charts in C1-C4 compare numbers of ED-1 + activated microglia between the two groups in the DG (**C1**), CA1 subfield (**C2**), CA3 subfield (**C3**) and the entire hippocampus (**C4**). Note that, RESV treatment significantly reduced numbers of ED-1 + activated microglia in the CA1 subfield (**A3,B3,C2**) and when the hippocampus was taken as a whole (**A1,B1,C4**). *p < 0.05; **p < 0.01.

Analyses using ELISA showed no changes in the concentration of pro-inflammatory cytokines TNF-α and IL-6 at 1-day after SE in SE-VEH and SE-RESV animals, in comparison to naive control animals (Fig. 7(A1). At 4-days post-SE, TNF-α protein concentration was increased in SE-VEH animals (p <  0.05, Fig. 7(A3)) but SE-RESV animals displayed concentrations comparable to those in naive control rats (p > 0.05, Fig. 7(A3)). However, IL-6 concentrations remained similar between groups at 4-days post-SE (Fig. 7(A4)). Analyses using ED-1 immunohistochemistry at 4-days post-SE revealed the presence of abundant activated microglia in the hippocampus of SE-VEH animals (Fig. 8(A1–A4)). However, SE-RESV animals demonstrated clearly reduced density of ED-1+ activated microglia in the hippocampus (Fig. 8(B1–B4)). Stereological quantification of ED-1+ cells confirmed a clear reduction in the number of ED-1+ activated microglia in SE-RESV animals (Fig. 8(C1–C4)). Although the reductions were modest in the DG (13% reduction, p > 0.05, Fig. 8(C1)) and the CA3 subfield (20% reduction, p > 0.05, Fig. 8(C3)), it was considerable for the CA1 subfield (46% reduction, p < 0.01, Fig. 8(C2)). When the hippocampus was taken in its entirety, SE-RESV animals displayed significantly reduced numbers of activated microglia (30% reduction, p < 0.05, Fig. 8(C4)). Thus, 4 days of RESV treatment prevented increases in TNF-α protein concentration as well as reduced the numbers of activated microglia.

### SE or RESV treatment after SE did not alter the expression of select genes related to inflammation, longevity and cognition

Measurement of genes related to inflammation in the hippocampus at 1-day after SE revealed no differences in the expression of IL-1β, NF-kB, IFN-γ, IL-4, IL-10 and MPO between SE-VEH, SE-RESV and naive control rats (p > 0.05, Table 1). The expression of TNF-α gene was enhanced in both SE-VEH and SE-RESV animals in comparison to naive control animals at this time point (p < 0.001, Table 1). No changes were however observed in the expression of any of the above 7 genes when examined at 4-days post-SE (Table 2). Animals receiving RESV during and after SE also did not display any increase in the expression of the longevity gene SIRT1 or FOXO3, a gene whose expression is dependent on SIRT1 activity and is important for cognitive function and synaptic plasticity. This was evidenced through similar expression of SIRT1 and FOXO3 between naive control animals and animals receiving VEH or RESV after SE when quantified at 1- and 4-days post-SE (p > 0.05, Tables 1 and 2). Thus, RESV mediated neuroprotection against SE did not seem to involve major modulation of the expression of genes related to inflammation, longevity or cognition.

## Discussion

This study provides the first evidence that RESV treatment commencing an hour after SE onset is efficient for protecting glutamatergic neurons in different hippocampal subfields, reducing the loss of several subclasses of GABA-ergic interneurons in the DG, and stemming anomalous hippocampal neurogenesis. Furthermore, RESV treatment mediated restrained neurodegeneration was allied with normalization of seizure-induced increased oxidative stress and modulation of inflammation, and diminished abnormal neurogenesis was accompanied with the preservation of reelin + interneurons in the DH. Because substantial interneuron loss, incessant oxidative stress and inflammatory activity, and aberrant neurogenesis are believed to be key contributors to epileptogenic processes that ensue after SE, these outcomes have great value towards developing a therapeutic regimen that curbs the progression of initial hippocampal injury into chronic TLE.

Status epilepticus emerges when sustained activation of glutamate receptors occurs in hippocampal principal neurons. Such stimulation primarily triggers prolonged hyperactivity of these neurons and then encompasses additional neuronal populations^38^. Synchronized and protracted hyperactivity leads to seizures accompanied with increased oxidative stress within neurons^39^. This ultimately initiates excitotoxic neuronal death in hippocampal regions such as DH comprising mainly distinct subpopulations of GABA-ergic interneurons and CA1 and CA3 cell layers filled mostly with glutamatergic pyramidal neurons. While the foremost neuron loss may cease with SE extinction, changes such as oxidative stress linger for extended periods after SE^40^. Both neurodegeneration and oxidative stress cause considerable inflammation with elevated levels of pro-inflammatory cytokines and activation of microglia^41^. Because all of these changes can participate in the evolution of initial SE-induced injury into chronic TLE, an ideal therapeutic strategy for SE therefore should be capable of protecting both principal and GABA-ergic neurons through suppression of oxidative stress and inflammation. It has been apparent from multiple studies that AEDs cannot accomplish these beneficial effects in spite of their ability to stop SE in many conditions^42^. The results of this study however demonstrate that RESV administration after SE has considerable promise for providing these beneficial effects.

The amount of neuroprotection seen with RESV administration was robust in this study. This was demonstrated by no significant neuron loss in the GCL, DH, and CA1 and CA3 pyramidal cell layers of SE-RESV animals, in contrast to neuron loss reaching 21-53% in these regions of SE-VEH animals. Moreover, SE-RESV animals displayed greater level of preservation of subclasses of GABA-ergic interneurons expressing PV, SST and NPY. This was evident from 30-37% greater numbers of these interneurons in the DG of SE-RESV animals at 4 days after SE, in comparison to the DG of SE-VEH animals. Although RESV treatment mediated interneuron protection is partial, this has considerable importance as studies have shown that severe loss of interneurons expressing calcium-binding proteins (e.g. PV + interneurons) and/or neuropeptides (e.g. SS + and NPY + interneurons) leads to chronic TLE exemplified by robust spontaneous recurrent seizures and cognitive and mood impairments^12^,^43^,^44^,^45^,^46^. The potential of RESV as a neuroprotective compound has been known from studies in several other neurological disease models. However, its ability for neuroprotection when applied after SE onset was unknown as previous studies in animal models were focused on examining the neuroprotective properties of RESV when administered prior to SE induction or after focal hippocampal injury induced under anesthesia^20^,^22^,^26^,^31^,^47^. The present study, by directly addressing this issue, however provided compelling evidence that RESV is neuroprotective even when applied after the onset of full-blown SE. Furthermore, RESV treatment did not seem to have anti-seizure effects, as animals treated with VEH or RESV displayed similar numbers of stages IV-V behavioral seizures in the first hour after treatment (i.e. in the second hour after SE onset). However, this finding needs detailed evaluation in the future studies using continuous EEG recordings after VEH or RESV treatment as well as following SE termination with diazepam injection. Such EEG studies would also be useful to detect the potential interactions between diazepam and RESV on seizure termination because a previous study has reported that higher doses of RESV can inhibit CYP3A4, a metabolic enzyme capable of increasing the bioavailability of diazepam^48^.

Nonetheless, analyses of biochemical, molecular and cellular changes in the hippocampus suggest that containment of SE-induced upsurge in oxidative stress and modulation of inflammation underlie RESV mediated neuroprotection. This is evinced by the following observations. Concerning oxidative stress, SE-RESV animals displayed MDA and 4-HNE (markers of lipid peroxidation) in the hippocampus to levels seen in SE-VEH animals when examined a day after SE, suggesting that RESV treatment for a brief period is insufficient for normalizing oxidative stress. However, 4 days after SE, RESV treated rats maintained hippocampal MDA to levels found in naïve control animals whereas SE-VEH animals sustained increased levels of MDA. Furthermore, the expression of many genes that classically exhibit upregulated expression in response to mounted oxidative stress was stabilized to control levels with RESV treatment. These comprise genes encoding proteins that are important for: (i) recruiting leukocytes into inflammatory sites (CCL5); (ii) detoxification of peroxides (GPX1, GPX7); (iii) reducing hydrogen peroxide, alkyl hydroperoxides (PRDX2, PRDX4) and intracellular nitric oxide (TXN1); (iv) promoting redox-sensitive chaperone activity (PARK7); (v) degradation of misfolded endoplasmic reticulum proteins (VIMP); (vi) reducing superoxide radicals (SOD1); and (vii) synthesizing glutathione (GCLC). The various other genes which respond to oxidative stress also showed a drift towards diminished expression in RESV treated rats, which include CAT, NUDT1, IDH1, PRDX1, SERPINB 1B, SEPP1, FTH1, APC and APOE. Intriguingly, RESV treatment after SE reduced the expression of some oxidative stress response genes (GSTP1, SOD2, GSR, IFT172, FANCC) to levels lesser than in naive control animals. Taken together, these observations underscore that 4 days of RESV treatment after SE is adequate for effecting oxidative stress to normal levels.

Restraining oxidative stress after SE has enormous value because increased oxidative stress has been shown to promote chronic neurodegeneration and dysfunction of surviving neurons in many neurological diseases, and elevated concentration of MDA is indeed seen in epileptic patients^40^,^49^. Normalization of oxidative stress by RESV treatment detected here is consistent with findings in other disease models using RESV^50^,^51^. However, RESV treatment for SE is likely to be effective only when oxidative stress is one of the prominent initial outcomes of SE. This is evident from an acute seizure study using neonatal animals, where no benefit of RESV treatment was observed on SE-induced neurodegeneration because SE in neonatal animals is not associated with increased oxidative stress^52^. Thus, RESV treatment for SE may be more suitable for the adult and aged populations where oxidative stress is one of the major initial pathological alterations.

The modulation of inflammation by RESV treatment was mainly evident from regulation of TNF-α protein concentration to control levels and reduced numbers of activated microglia at 4 days after SE. However, examination of the expression of select genes encoding pro-inflammatory and antiinflammatory cytokines and NF-κB (a regulator of inflammation^53^,^54^) did not reveal significant alterations between groups except for TNF-α gene, which showed increased expression in both SE-VEH and SE-RESV animals a day after SE. Likewise, SE-RESV animals did not exhibit activation of the longevity gene SIRT1 or its downstream target FOXO3 likely due to a shorter duration and lower dose of RESV treatment. Thus, RESV-mediated suppression of SE-induced inflammation did not involve modulation of NF-κB or SIRT1 activity. Considering these, the likely mechanism by which RESV modulated inflammation is via repression of oxidative stress after SE, which greatly reduced neuronal loss and hence severe inflammation did not ensue in SE-RESV animals. Regardless of the mode of action, reduced inflammation observed after SE with RESV intervention has implications because persistent inflammation is one of the key players in the evolution of neurodegenerative diseases including TLE^55^. For instance, increased levels of pro-inflammatory cytokines are consistently seen in the epileptic brain^56^. The sources of these pro-inflammatory cytokines in the brain are activated microglia and reactive astrocytes. Microglia cells are the resident macrophages in the brain, and in normal conditions, they are known to probe their environment^57^. When they sense injury signals, they get activated and release pro-inflammatory cytokines^56^. Thus, normalization of TNF-α concentration and reduced numbers of ED-1+ activated microglia in SE-RESV animals imply that RESV administration is effective for restraining SE-induced inflammation.

Another exciting aspect of RESV treatment mediated benefits after SE was its ability to restrain abnormal neurogenesis. Abnormal neurogenesis, typified by aberrant migration of newly born neurons into the DH and occurrences of basal dendrites from neurons that incorporate into the GCL, is a common phenomenon after SE^58^. Indeed, abnormal neurogenesis was apparent in SE-VEH animals but clearly reduced in SE-RESV animals. Suppression of SE-induced abnormal neurogenesis through RESV administration has significance because multiple studies have shown that aberrant neurogenesis promotes the development of epileptogenic circuitry between granule cells displaced into the DH and CA3 pyramidal neurons, and between basal dendrites of granule cells projecting into the DH and granule cell axons. Although conclusive evidence showing that these abnormalities alone are sufficient to cause chronic TLE is lacking^59^, their contribution towards occurrences of spontaneous recurrent seizures in the chronic phase after SE has been well recognized^37^,^60^,^61^,^62^,^63^,^64^,^65^. To understand the mechanism underlying RESV-mediated suppression of abnormal neurogenesis, we quantified reelin + interneurons in the DH. This is because, a previous study has demonstrated that migration of DCX + newly born neurons from the SGZ to GCL is guided by reelin protein secreted by reelin + interneurons in the DH and substantial loss of reelin producing neurons can promote abnormal migration of newly born neurons into the DH^66^. Our analysis confirmed the occurrence of abnormal neurogenesis in association with substantial loss of reelin + interneurons in SE-VEH animals and reduced extent of abnormal neurogenesis with preservation of reelin + interneurons in SE-RESV animals. Thus, RESV administration restrained abnormal neurogenesis through preservation of reelin + interneurons and this protection is likely a result of suppression of oxidative stress and inflammation mediated by RESV.

Examination of the extent of normal neurogenesis in the SGZ-GCL region did not show apparent differences between SE-VEH and SE-RESV animals. Our recent study using aged animals has however shown increased normal neurogenesis with four weeks of RESV treatment^67^. Lack of increase in normal neurogenesis with RESV treatment in the current study likely reflects a shorter duration of RESV administration (4 days) vis-à-vis 4 weeks of RESV treatment employed in our previous study^67^.

## Conclusion and Future Studies

This study provides novel evidence that RESV administration commencing after SE onset is highly beneficial for restraining SE-induced oxidative stress, neurodegeneration, neuroinflammation and abnormal neurogenesis, all of which have been recognized as epileptogenic changes or precursors of chronic epilepsy development. Considering these, RESV administration seems suitable as an adjunct to AED therapy for SE. In such scenario, a suitable combination of AEDs can help in terminating SE emergency whereas RESV administration for extended periods after SE would help in modulating the SE-induced oxidative stress and neuroinflammation, which may lessen the propensity of SE incidence from progressing into a chronic epileptic state. However, follow-up studies will be needed in the future to determine whether the extent of neuroprotection provided by RESV administration against SE-induced oxidative stress and inflammation is sufficient to prevent or greatly restrain the evolution of SE-induced hippocampal injury into chronic epilepsy development typified by SRS and cognitive and mood dysfunction. Furthermore, the required dose and duration of RESV treatment after SE to prevent chronic epilepsy development will need to be addressed.

## Methods

### Animals

Young adult (3-4 months old) male F344 rats, obtained from Harlan, were used in this study. Animals were housed in an environmentally controlled room with a 12:12-hr light-dark cycle and were given food and water ad libitum. All experiments were performed as per the animal protocol, approved by the institutional animal care and use committee of the Texas A&M Health Sciences Center and Central Texas Veterans Health Care System.

### Induction of SE and group assignment

After 7-10 days of acclimatization, SE was induced in 66 rats through graded intraperitoneal injections of kainic acid (KA, 2.5-5.0 mg/Kg) every hour until they displayed either a state of continuous stage IV seizures characterized by bilateral forelimb clonus with signs of rearing, or a first stage V seizure typified by bilateral forelimb clonus with rearing and falling followed by continuous stages III-V seizures for over 10 minutes^12^,^34^,^35^,^36^. Animals were allowed to have multiple stages III-V seizures for an hour after the onset of SE and then assigned randomly to RESV or VEH groups. The behavioral seizures in both VEH and RESV treated groups were terminated 2 hours after the induction of SE through an intraperitoneal injection of diazepam (5 mg/Kg). This SE prototype has 20–30% mortality occurring either during SE (due to uncontrolled bouncing and/or tonic-clonic seizures) or in the first few days after SE when 3-4 months old rats are employed. Furthermore, 10–20% of rats do not meet all SE criteria despite receiving 4-5 injections of KA and such animals were excluded from the study. A total of 36 rats met SE criteria and survived the entire duration of the experiment in this study.

### Treatment regimen and animal numbers utilized for various analyses

Animals received intraperitoneal injections of vehicle (VEH, n = 17) or RESV (n = 19, 30 mg/Kg b.w.) after SE, which commenced an hour after the onset of SE and continued hourly for three hours on SE day and twice daily thereafter for the next 3 days. Subsets of rats were euthanized a day after SE (VEH, n = 4; RESV, n = 6) and 4-days after SE (VEH, n = 4; RESV, n = 5) with deep anesthesia followed by decapitation. Fresh brain tissues from these animals were harvested for biochemical and molecular biological studies. Brain tissues from a group of age-matched naive control rats (n = 6) were also harvested similarly for comparison. Additional subsets of rats from both SE groups (VEH, n = 9; RESV, n = 8) were perfused 4 days after SE for immunohistochemical processing of hippocampal tissue sections and quantification of the extent of neurodegeneration, aberrant neurogenesis and neuroinflammation. Hippocampal tissue sections harvested earlier from a group of age-matched naive control rats (n = 6) were also processed for immunohistochemistry for comparison.

### Tissue processing and immunohistochemistry

Each animal belonging to SE-VEH and SE-RESV groups (n = 8-9/group) was deeply anesthetized through isoflurane vapor exposure and then perfused through the heart using 4% paraformaldehyde. Animal perfusion and tissue processing protocols are detailed in our previous reports^12^,^34^. Thirty-micrometer thick cryostat sections were cut coronally through the entire septo-temporal axis of the hippocampus and collected serially in 24-well plates containing phosphate buffer (PB). Serial sections (every 20th) through the entire hippocampus were selected in each of the animals belonging to SE-VEH and SE-RESV groups and processed for immunohistochemical studies, along with tissue sections of similar thickness and comparable levels obtained from age-matched naive control animals (n = 6). The studies comprised detection of: (i) neuron-specific nuclear antigen (NeuN, a marker of all mature neurons); (ii) markers of subclasses of gamma-amino butyric acid (GABA) positive interneurons such as neuropeptide Y (NPY), somatostatin (SST), parvalbumin (PV) and reelin; (iii) ED-1 (a marker of activated microglia) and (iv) doublecortin (DCX, a marker of newly born neurons). The detailed procedures employed for immunohistochemical staining for the above markers are described in our previous reports^1^,^12^,^68^,^69^,^70^,^71^,^72^. In brief, sections were first treated with phosphate buffered saline (PBS) solution containing 20% methanol and 3% hydrogen peroxide for 20 minutes and then rinsed thrice in PBS. Next, the sections were treated for 30 minutes in PBS containing 0.1% Triton-X 100 and an appropriate serum (10%) selected on the basis of the species in which the chosen secondary antibody was raised. Sections were then incubated 18-48 hours in primary antibody solutions prepared in PBS. The primary antibodies comprised mouse monoclonal anti-NeuN (1:1000, Millipore), anti-parvalbumin (1:2000, Sigma), anti Reelin (1:1000, Millipore), and anti ED-1 (1:1000, Serotech), rabbit monoclonal anti-NPY (1:10,000, Peninsula laboratories) and anti-SST (1:5000, Calbiochem), and goat polyclonal anti-DCX (1:200; Santa Cruz Biotechnology). Following primary antibody incubation, sections were washed thrice in PBS, incubated in an appropriate biotinylated secondary antibody (anti-goat, anti-mouse or anti-rabbit IgG, Vector Laboratories) solution for 60 minutes, washed thrice in PBS and treated with avidin-biotin complex reagent (ABC, Vector) for 60 minutes. The peroxidase reaction was then developed using diaminobenzidine (DAB, Vector). The sections were mounted on gelatin coated slides, dehydrated, cleared and cover slipped.

### Stereological quantification of neurons, interneurons, activated microglia and newly born neurons in the hippocampus

The optical fractionator method in the StereoInvestigator system (Microbrightfield Inc., Williston, VT) interfaced with a Nikon E600 microscope through a color digital video camera (Optronics Inc., Muskogee, OK) was employed for all cell counts. It comprised counting of: (i) NeuN + mature neurons in the dentate hilus (DH), the granule cell layer (GCL), and CA1 and CA3 pyramidal cell layers; (ii) NPY-, SST-, PV- and reelin-positive interneurons in the dentate gyrus (DG); (iii) ED-1+ activated microglia in the DG, CA1 and CA3 subfields of the hippocampus; and (iv) DCX + newly born neurons in the DH. Every 20th section through the entire septo-temporal axis of the hippocampus from each animal belonging to RESV, VEH and naive control groups (n = 6/group) was employed for this quantification. The methodology utilized for the optical fractionator stereological counting is detailed in our earlier reports^12^,^34^,^68^,^69^,^70^. In brief, using a 100X lens, cells were counted in every 20th section from 50-500 frames (each measuring 40 × 40 μm) selected through a systematic random sampling scheme. The contour of the chosen hippocampal area was first demarcated in every section using the tracing function. Next, by entering parameters such as grid size, thickness of the top guard zone (4 μm) and the optical dissector height (i.e. 8 μm), numbers and locations of counting frames and the depth for counting were determined. A computer steered motor-powered stage then permitted the section to be evaluated at every counting frame sites. All cells (expressing NeuN, NPY, SST, PV, reelin, ED-1 or DCX) that were present within the 8 μm section depths in each site were counted if they were entirely within the counting frame or touching the upper or right side of the counting frame. This process was repeated for all sections. A choice in the Stereo Investigator program let the experimenter to remain oblivious to the running cell counts until all sections for each animal were completed. The StereoInvestigator program later calculated the total number of cells in each chosen region by utilizing the optical fractionator formula, as described in our earlier reports^12^,^68^.

### Tissue processing for biochemical and molecular biology studies

Biochemical and molecular biological studies of hippocampal tissues were performed at one-day and 4-days after the induction of SE. For these, each animal belonging to SE-VEH and SE-RESV groups (n = 4–6/time-point/group) and an age-matched naive control group (n = 6) was euthanized through decapitation following deep anesthesia with isoflurane vapor exposure. The brain was rapidly dissected, snap frozen using dry ice and stored at minus 80 °C. Once the required numbers of brains from all groups are collected and stored for weeks, the entire hippocampus from each side of every brain was micro-dissected following thawing. Each hippocampus was then cut coronally into six pieces of approximately comparable size along its antero-posterior axis. The odd numbered tissue pieces (1, 3 and 5) were processed for biochemical assays and the even numbered tissue pieces (2, 4 and 6) were processed for molecular biological studies. The biochemical studies comprised: (i) quantification of natural byproducts of lipid peroxidation using malondialdehyde (MDA) assay and competitive enzyme-linked immunoassay (ELISA) for 4-hydroxynonenal (4-HNE); and (ii) ELISAs for pro-inflammatory cytokines such as tumor necrosis factor-alpha (TNFα) and interleukin-6 (IL-6). The molecular biological studies comprised analyses of the expression of genes encoding oxidative stress, antioxidant proteins, pro-inflammatory cytokines and antiinflammatory cytokines, and genes important for longevity and synaptic plasticity, using quantitative real time polymerase chain reaction (qRT-PCR).

### Measurement of oxidative stress markers in the hippocampus

The lysate from the hippocampus of each animal was prepared through homogenization in 750 μl of tissue extraction reagent (Life Technologies, Grand Island, NY) with protease inhibitors (Sigma-Aldrich Corp. St. Louis, MO) using a sonic dismembrator for 10 seconds. The lysates were centrifuged twice at 12,000 g for 10 minutes at 4 °C and aliquots of the collected supernatant were stored at -80 °C until used. The extent of oxidative stress in the hippocampus was ascertained through measurement of malondialdehyde (MDA) and 4-hydroxy-2-nonenal (4-HNE) concentrations, 1-day and/or 4-days after SE. MDA is a byproduct of lipid peroxidation and an important biomarker of membrane damage. On the other hand, 4-HNE is α, β-unsaturated hydroxyalkenal produced by lipid peroxidation in cells. For MDA analysis, aliquots of tissue lysates were processed in duplicates as per the manufacturer’s protocol for “TBARS Assay Kit” (Cayman Chemical Company, MI, USA). Briefly, 100 μl of sample was mixed with an equal amount of SDS solution and 4 ml of a color reagent containing thiobarbituric acid in a tube and boiled for 1 hour using a water bath. The tubes were then cooled and samples were centrifuged at 1,600 g at 4 °C. The supernatant obtained was loaded on colorimetric plate with 150 μl in each well and absorbance was read at 540 nm. The MDA concentration was then determined in brain samples using a calibration curve prepared from standard MDA processed similarly and expressed as nanomoles/mg of brain tissue. The HNE protein adducts were measured by following the manufacturer protocol for “Oxiselect^TM^ HNE adduct competitive ELISA kit” (Cell Biolabs, CA, USA). Briefly, 50 μl of tissue aliquot and HNE BSA standard were added to the wells of HNE conjugate coated plate and incubated at room temperature for 10 minutes with shaking. After incubation, 50 μl of diluted anti-HNE antibody supplied along with kit was added to each well of the plate and incubated at room temperature for an hour. Next, wells were washed thrice with wash buffer and incubated with 100 μl of diluted secondary antibody conjugated to HRP for an hour. Following this, wells were loaded with 100 μl of the substrate solution, incubated for 10 minutes and the reaction was stopped by the addition of 100 μl of stop solution to each well. The absorbance was read immediately at 450 nm. The assay was performed in duplicates for each sample and concentrations thus obtained were expressed as μg/mg of tissue.

### Analyses of the expression of genes related to oxidative stress

Hippocampal samples from VEH and RESV treated groups collected 4 days after SE were processed for “The Rat Oxidative Stress PCR Array” from Qiagen, along with samples from age-matched naive control animals (n = 4/group). The total RNA was first extracted using RNeasy kit (Qiagen, Valencia, CA) by following manufacturer’s instructions and methods detailed in our previous report^71^. The RNA concentration (A260) and quality (A260:A280) were determined by Nanodrop spectrophotometer (Thermo Scientific, Wilmington, DE). Total RNA (1 μg) was subsequently transcribed to cDNA using the “RT2 First Strand Kit” (Qiagen, Valencia, CA) as per manufacturer’s protocol and our previous study^73^. The Rat Oxidative Stress PCR Array from Qiagen profiles the expression of 84 key genes that are relevant for reactive oxygen species (ROS) metabolism, oxygen transporters and antioxidants. The reactions were done as per manufacturer’s protocol using a CFX96 Real-Time system (Bio-Rad, Hercules, CA). The PCR amplification was followed by a melt curve analysis to evaluate the specificity of the reaction. The cycling conditions were as described in our previous study^73^ and the Ct (threshold cycle) values of all wells were exported to Excel spreadsheet and analyzed using web based SABiosciences PCR array data analysis software. 2^delta Ct values for each gene from different groups were statistically compared.

### Measurement of pro-inflammatory cytokines TNF-α and IL-6

Levels of TNFα and IL-6 proteins were quantitated using rat solid phase sandwich ELISA kits (Invitrogen, Carlsburg, CA) using the manufacturer’s protocol. Briefly, the diluted samples, known standards and controls were incubated on wells of the microtiter strips that were pre-coated with a specific monoclonal antibody. The wells were washed and then incubated with a biotinylated secondary antibody. Streptavidin-peroxidase enzyme was added to the washed wells to bind to the biotinylated secondary antibody. After removing the unbound enzyme through washing, a substrate solution was added. The peroxidase reaction produced a color, with an intensity that is directly proportional to the respective concentration of TNFα or IL-6 present in the sample.

### Analyses of the expression of genes related to inflammation, longevity and cognition

Hippocampus samples from VEH and RESV treated groups collected 1 and 4 days after SE were also used for measuring the expression of select genes related to inflammation, longevity and cognition, along with samples from age-matched naive control animals (n = 4-6/group). The total RNA and cDNA were prepared as described above. The template cDNA was next amplified separately using specific primers (Qiagen, Valencia, CA) of multiple genes. This comprised genes linked to inflammation (interleukin-1beta [IL-1β], TNFα, nuclear factor kappa B [NFκB], interferon-gamma [IFN-γ], IL-4 and IL-10, myeloperoxidase [MPO]), and longevity and cognition (NAD-dependent deacetylase sirtuin-1 [SIRT1], Forkhead box O3 [FOXO3]). In brief, for each qPCR reaction, 1 μl template cDNA was mixed with 12.5 μl of 2X SYBR master-mix (SABiosciences, Qiagen, Valencia, CA), 1 μl of gene specific primer (RT2 qPCR primer mix containing 10 μM each of forward and reverse primers, Qiagen, Valencia, CA) and 10.5 μl of dH2O for a total volume of 25 μl. Each assay also comprised two housekeeping genes namely GAPDH and Act B. After a brief centrifugation, reactions were carried out using a CFX96 Real-Time system (Bio-Rad, Hercules, CA). Data collection and analyses were performed as described above.

### Statistical Analyses

Statistical analyses were performed using one-way analyses of variance (one-way ANOVA) with Newman-Keuls multiple comparison post hoc tests when comparisons involved three or more groups. When comparisons involved only two groups, unpaired, two-tailed Student’s-t test was employed. Data were expressed as means ± SEM and a p value less than 0.05 was considered as statistically significant.

## Additional Information

**How to cite this article**: Mishra, V. *et al*. Resveratrol Treatment after Status Epilepticus Restrains Neurodegeneration and Abnormal Neurogenesis with Suppression of Oxidative Stress and Inflammation. *Sci. Rep*. **5**, 17807; doi: 10.1038/srep17807 (2015).

## Figures and Tables

**Table 1:** Expression of Select Genes Related to Inflammation, Longevity and Cognition when examined a day after status epilepticus (SE).

Gene	Naïve Control	SE-VEH	SE-RESV	ANOVA
	Mean ± S.E.M	Mean ± S.E.M	Mean ± S.E.M	p-value
IL1β	0.000220 ± 0.00002	0.000313 ± 0.000047	0.0002425 ± 0.000019	p > 0.05
TNF-α	0.000203 ± 0.00007	0.00070 ± 0.000038	0.00058 ± 0.000046	p < 0.0001
(Naive vs SE-VEH, p < 0.001; Naive vs SE-RESV, p < 0.001, SE-VEH vs SE-RESV, p > 0.05)				
NF-kB	0.00476 ± 0.00098	0.009995 ± 0.0011	0.007802 ± 0.00162	p > 0.05
IFN-γ	0.00002 ± 0.000002	0.000014 ± 0.000002	0.0000115 ± 0.0000007	p > 0.05
IL4	0.000084 ± 0.000009	0.00017 ± 0.0000077	0.00018 ± 0.000034	p > 0.05
IL10	0.003974 ± 0.001234	0.00263 ± 0.000555	0.00360 ± 0.00048	p > 0.05
MPO	0.000035 ± 0.000009	0.000121 ± 0.000039	0.000085 ± 0.0000145	p > 0.05
SIRT1	0.00745 ± 0.0007	0.008357 ± 0.0007	0.008581 ± 0.00071	p > 0.05
FOXO3	0.01386 ± 0.0018	0.01015 ± 0.0008	0.01125 ± 0.00072	p > 0.05

The numbers depict 2^delta Ct values. IL1β, interleukin 1-beta; TNF-α, tumor necrosis factor-alpha; NF-kB, nuclear factor of kappa light polypeptide gene enhancer in B-cells; IFN-γ, interferon-gamma; MPO, myeloperoxidase; IL4, interleukin 4; IL10, interleukin 10; SIRT1, sirtuin 1; FOXO3, forkhead box O3.

**Table 2:** Expression of Select Genes Related to Inflammation, Longevity and Cognition when examined four days after status epilepticus (SE).

Gene	Naïve Control	SE-VEH	SE-RESV	ANOVA
	Mean ± S.E.M	Mean ± S.E.M	Mean ± S.E.M	p-value
IL1β	0.000220 ± 0.00002	0.000155 ± 0.000032	0.0001954 ± 0.000056	p > 0.05
TNF-α	0.000203 ± 0.00007	0.000304 ± 0.000046	0.0002614 ± 0.000025	p > 0.05
NF-kB	0.00476 ± 0.00098	0.005360 ± 0.000206	0.005769 ± 0.000647	p > 0.05
IFN-γ	0.00002 ± 0.000002	0.005914 ± 0.003413	0.005713 ± 0.0035	p > 0.05
IL4	0.000084 ± 0.000009	0.000066 ± 0.000018	0.000052 ± 0.000005	p > 0.05
IL10	0.003974 ± 0.001234	0.000376 ± 0.000340	0.001913 ± 0.000865	p > 0.05
MPO	0.000035 ± 0.000009	0.000032 ± 0.000009	0.000022 ± 0.000004	p > 0.05
SIRT1	0.00745 ± 0.0007	0.005498 ± 0.000476	0.005553 ± 0.00049	p > 0.05
FOXO3	0.01386 ± 0.0018	0.00565 ± 0.003343	0.008032 ± 0.00358	p > 0.05

The numbers depict 2^delta Ct values. IL1β, interleukin 1-beta; TNF-α, tumor necrosis factor-alpha; NF-kB, nuclear factor of kappa light polypeptide gene enhancer in B-cells; IFN-γ, interferon-gamma; MPO, myeloperoxidase; IL4, interleukin 4; IL10, interleukin 10; SIRT1, sirtuin 1; FOXO3, forkhead box O3.
